# Electrophysiological assessment of auditory processing disorder in children with non-syndromic cleft lip and/or palate

**DOI:** 10.7717/peerj.2383

**Published:** 2016-08-25

**Authors:** Xiaoran Ma, Bradley McPherson, Lian Ma

**Affiliations:** 1 Division of Speech and Hearing Sciences, University of Hong Kong, Hong Kong, China; 2 School of Stomatology, Peking University, Beijing, China

**Keywords:** Auditory brainstem responses, Cleft palate, School age children, Auditory processing disorder, Cleft lip

## Abstract

**Objectives:**

Cleft lip and/or palate is a common congenital craniofacial malformation found worldwide. A frequently associated disorder is conductive hearing loss, and this disorder has been thoroughly investigated in children with non-syndromic cleft lip and/or palate (NSCL/P). However, analysis of auditory processing function is rarely reported for this population, although this issue should not be ignored since abnormal auditory cortical structures have been found in populations with cleft disorders. The present study utilized electrophysiological tests to assess the auditory status of a large group of children with NSCL/P, and investigated whether this group had less robust central auditory processing abilities compared to craniofacially normal children.

**Methods:**

146 children with NSCL/P who had normal peripheral hearing thresholds, and 60 craniofacially normal children aged from 6 to 15 years, were recruited. Electrophysiological tests, including auditory brainstem response (ABR), P1-N1-P2 complex, and P300 component recording, were conducted.

**Results:**

ABR and N1 wave latencies were significantly prolonged in children with NSCL/P. An atypical developmental trend was found for long latency potentials in children with cleft compared to control group children. Children with unilateral cleft lip and palate showed a greater level of abnormal results compared with other cleft subgroups, whereas the cleft lip subgroup had the most robust responses for all tests.

**Conclusion:**

Children with NSCL/P may have slower than normal neural transmission times between the peripheral auditory nerve and brainstem. Possible delayed development of myelination and synaptogenesis may also influence auditory processing function in this population. Present research outcomes were consistent with previous, smaller sample size, electrophysiological studies on infants and children with cleft lip/palate disorders. In view of the these findings, and reports of educational disadvantage associated with cleft disorders, further research that focuses on the auditory processing abilities of children with cleft lip/palate disorder is warranted.

## Introduction

Cleft lip and/or palate (CL/P) is a congenital craniofacial anomaly, contributing to human birth defects in all populations. The majority of individuals with CL/P are diagnosed with non-syndromic cleft lip and/or palate (NSCL/P), indicating a cleft disorder in isolation from other abnormal phenotypes (Stanier & Moore, 2004).

Peripheral hearing problems are common in children with NSCL/P, with a high prevalence of middle ear deficits often attributed directly or indirectly to Eustachian tube dysfunction (Bluestone & Doyle, 1988; Sheer, Swarts & Ghadiali, 2012). In western populations, prevalence figures for unilateral or bilateral conductive hearing loss of greater than 50% (Caldarelli, 1978) to 75% (Flynn et al., 2009) are frequently reported in children with CL/P. Reports indicate that this hearing deficit is generally bilateral, fluctuating, and mild to moderate in nature (Yang & McPherson, 2007). Increased levels of learning disability, reading disability, and academic under-achievement (Conrad et al., 2014; Persson, Becker & Svensson, 2012; Richman et al., 2012) have been associated with children with CL/P. Collett et al. (2010) found that scores for tasks involving reading and related skills were significantly lower in children with nonsyndromic clefts and suggested that this may be partly attributed to an increased prevalence of hearing disorders. However, several research groups (Conrad et al., 2014; Hubbard et al., 1985; Jocelyn, Penko & Rode, 1996) noted that a history of conductive hearing loss does not fully account for the extent of language and learning delays seen in children with CL/P.

Recently, auditory processing disorder (APD) has been flagged as an additional hearing deficit that may be prevalent in children with CL/P—potentially another contributing factor to language and learning delay. APD is a perceptual disorder thought to result from impaired brainstem/cortical function. As described by the American Speech-Language-Hearing Association (American Speech-Language-Hearing Association, 2005), individuals with APD frequently show deficits or poor performance in one or more of the following listening skills: sound localization and lateralization; auditory discrimination; auditory pattern recognition; temporal aspects of audition; auditory performance decrements with competing acoustic signals and degraded acoustic signals. Using behavioral assessments (Boscariol, André & Feniman, 2009; Ma, McPherson & Ma, 2015) and questionnaire appraisals (Ma, McPherson & Ma, 2016; Minardi et al., 2004) children with CL/P often have indications of poorer auditory processing abilities than their craniofacially normal peers, and this is the case even for children with NSCL/P who have no history of middle ear disorder.

Differences exist in cortical structure between individuals with NSCL/P and their craniofacially normal peers. Nopoulos et al. (2000), Nopoulos et al. (2002) and Nopoulos et al. (2006) found that young adult men with NSCL/P had radiologically abnormal cortical regions, with the most significant differences in the left temporal lobe, which showed reductions of both gray and white matter volume compared to craniofacially normal controls. Such differences may lead to altered functional capabilities in the auditory cortex. Decreased volume and thickness in the left superior temporal plane, and other cortical developmental anomalies, have been reported in infants with NSCL/P (Yang et al., 2012b).

Electrophysiological assessment offers a window on auditory function, with fewer requirements for cooperation from listeners compared to behavioral hearing tests. Furthermore, a series of distinct auditory evoked potential (AEP) peaks appearing after different latencies that presents neural activity from different anatomical stations along the auditory pathway supports diagnosis of hearing loss and helps to locate lesions in the auditory system (Eggermont, 2007). There is broad consensus that an APD diagnostic battery is not comprehensive if it does not include electrophysiological measures (Bellis, 2003a) because these tests are objective, do not require a patient response and carry less linguistic load than many behavioral tests (Bellis, 2003b; Liasis et al., 2003). For children with CL/P, reports on the application of AEPs to evaluate central auditory function are not common. Typically, utilization of auditory brainstem response (ABR) has predominantly been reported in this population since it is a reliable assessment tool to evaluate the peripheral auditory status of children. ABR has been used on children with CL/P before 12 months of age, and it was found most children with CL/P showed mild to moderate conductive hearing loss at an early age (Hélias et al., 1988). Moreover, a UK study indicated more than 80% of infants with cleft palate (CP) had abnormal ABR responses, and most noted hearing loss was conductive, mild and bilateral (Viswanathan, Vidler & Richard, 2008). Aside from ABR, a series of studies conducted by researchers from the University of Helsinki used mismatch negativity (MMN) as an index to compare auditory cortical function between children with CL/P (both non-syndromic CL/P and CL/P with syndrome) and craniofacially normal children (Ceponiene et al., 2002; Ceponiene et al., 2000; Ceponiene et al., 1999; Cheour et al., 1999; Cheour et al., 1997). In these studies, auditory memory span time, which refers to the time required for accurate recall of an acoustic input during auditory processing, was found to be shorter in children with CL/P than their craniofacially normal peers, despite the children with CL/P not having history of hearing disorder (Cheour et al., 1997). Also, infants with CP showed the most impaired MMN responses compared to infants with CLP and CL (Ceponiene et al., 2000; Ceponiene et al., 1999). More recently, a study also found significant group differences for MMN between infants with NSCL/P and normal peripheral hearing status and infants who were craniofacially normal (Yang et al., 2012a).

Although the electrophysiological research mentioned above has provided useful information regarding hearing abilities—from peripheral to cortical auditory function in children with CL/P—all studies used single electrophysiological tests for evaluation of APD-related functions on small numbers of infants. However, comprehensive assessment using both short latency evoked potentials and long latency responses, including obligatory potentials and later cortical responses, for a large sample of school age children with CL/P has not been reported. In the current study, short latency potentials (ABR), long latency P1-N1-P2 complex responses, as well as P300 components related to auditory attention and working memory, were recorded in children with NSCL/P who had both normal hearing thresholds and middle ear function at the time of assessment, and results were analyzed to determine cleft type and age effects in this clinical population compared to their craniofacially normal peers.

## Materials and Methods

### Participants

There were 146 children (292 ears) with NSCL/P aged from 6.00 to 15.67 years (mean = 10.08) recruited for electrophysiological assessment into the current study. All of the children (98 males and 48 females) were native Mandarin speakers and attended regular schools. They were divided into three subgroups by cleft type: 37 children with CL, 26 children with CP, and 83 children with CLP. The CLP group was further divided into unilateral CLP (UCLP) and bilateral CLP (BCLP) groups. Also, the children with NSCL/P were categorized by age group: 52 children aged from 6 to 8 years; 56 children aged from 9 to 11 years; and 38 children aged from 12 to 15 years. The children were visiting the outpatient department of the Cleft Lip and Palate Clinic, Beijing Stomatology Hospital, for further consultation, as recommended by their primary care doctors, typically for possible cleft lip/palate secondary repair surgery. A control group, comprised of 60 children who were craniofacially normal (25 boys and 35 girls) aged from 6.00 to 15.50 years (mean = 10.16), was also studied. Among them were 20 children aged from 6 to 8 years, 20 children aged from 9 to 11 years, and 20 children aged from 12 to 15 years. As with the NSCL/P group, all were native Mandarin speakers and attended regular schools. All children had normal hearing status at the time of assessment. Children were screened using a protocol that followed American Speech-Language-Hearing Association (1997) guidelines for screening for hearing impairment and middle ear disorders. The protocol included otoscopy, pure tone screening audiometry, 226 Hz probe tone tympanometry and an ipsilateral 1 kHz acoustic reflex threshold test. The procedure was conducted in a quiet research room, with ≤35 dB A ambient noise. Children with otoscopic abnormalities, who failed pure tone screening (>25 dB HL at a 0.5, 1, 2, and 4 kHz average, in either ear), who had other than type A tympanograms (based on Jerger, 1970, classification with Wan & Wong, 2002, Chinese norms, in either ear) or who did not present bilaterally with a 1 kHz ipsilateral reflex at ≤105 dB HL, were excluded from the study (*n* = 37; 20%). Participant numbers by age range and cleft status are shown in Table 1. Prior to participation in the research program, parent and student written consent was obtained. The study was approved by the Human Research Ethics Committee for Non-Clinical Faculties, The University of Hong Kong (reference number EA140811).

**Table 1:** Participant numbers by age range in NSCL/P and control groups.

	NSCL/P group				Total NSCL/P	Control group
	CL	CP	CLP			
			UCLP	BCLP		
6–8 years	14	13	17	8	52	20
9–11 years	11	9	22	14	56	20
12–15 years	12	4	16	6	38	20
Total	37	26	55	28	146	60

**Notes.**

TITLE CL Cleft Lip CP Cleft Palate CLP Cleft Lip and Plate UCLP Unilateral Cleft Lip and Palate BCLP Bilateral Cleft Lip and Plate NSCL/P Non-syndromic Cleft Lip and/or Palate

### Auditory evoked potentials recording

In the study, ABR testing was used to evaluate the integrity of the auditory pathway from the eighth nerve to brainstem level. The evaluation system was the Eclipse EP15 ABR platform (Interacoustics, Denmark). The acceptable electrode impedance value was 3 kΩ or lower. An electrode was placed at the high forehead (Fz) as the active electrode. Reference electrodes were placed on right and left mastoids and the midpoint between the eyebrows was used as ground.

Monaural alternating rarefaction click stimuli (100 µs duration) were presented via Ear Tone 3A ABR insert phones at a rate of 44.1 per second with a standard intensity of 80 dB HL. The evoked electrical signals were amplified and filtered (filter setting 100–3,000 Hz) with an averaging window of 20 ms and a sum of 2,000 sweeps in each averaged waveform. The sensitivity/artifact rejection level was set at ±40 µV. The waves were evaluated by considering the latencies of wave I, III, V and I–V interpeak latencies. The selection of the ABR wave latencies used in the study was due to the robust nature of the signal, as they are virtually unaffected by different positions of the recording electrodes (Don & Kwong, 2009). In addition, the interpeak latency interval (IPL) from wave I to wave V is recommended for use to evaluate the interaural latency difference (ILD) (Moller & Moller, 1985). The ABR recording protocol was based on standard settings for children used in previous studies (Sininger, 1993; Stueve & O’Rourke, 2003). Table 2 shows the detailed parameter settings for the ABR recording protocol.

**Table 2:** ABR recording protocol: parameter settings.

Parameter	Setting
Stimulus	100 µs click
Rate	44.1/s
Polarity	Alternating
Transducers	Insert phones (Ear Tone 3A)
Intensity	80 dB nHL
Filters	100–3,000 Hz
Analysis time window	0–20 ms
Sweeps	2,000
Sensitivity/artifact reject	±40 µV

The P1-N1-P2 complex and P300 were recorded to evaluate the ability of participants to detect acoustic changes before sound discrimination and conscious post-decision discrimination related to attention and memory, respectively. These late evoked potentials (LEPs) were recorded utilizing the same equipment as for the ABR test, and the electrode placement was also the same as that used with the ABR assessment. The stimuli used to elicit obligatory AEPs and P300 were deviant (i.e., infrequent) tones embedded in a series of standard (i.e., frequent) stimuli. The placement of the rare sounds within the series of frequent sounds was random. The standard tone stimuli were 1 kHz with an intensity of 60 dB HL, and appeared 80% of the time. Deviant tones were at 2 kHz with an intensity of 90 dB HL, and occurred 20% of the time. Generally, the amplitudes and latencies of these LEPs depend on the magnitude of the difference between the standard and deviant stimuli, and more robust signals can be elicited by using a larger difference (Liasis et al., 2003). The selection of stimuli intensities in the current study was based on established procedures reported in the literature (Cone-Wesson & Wunderlich, 2003; Stapells, 2009). A key difference with ABR recording is that LEP tests require the listener to actively attend to the infrequent sounds. This attentional process typically involves counting the number of the rare stimuli presented, and this procedure was used in the present study.

N1 amplitude changes with stimulus duration and rise-fall times, and decreases if the stimulus duration is longer than 30 ms and the rise-fall times are longer than 50 ms (Alain & Woods, 1997; Onishi & Davis, 1968). In the current study, the stimuli were tone bursts with 20 ms rise-fall times, 20 ms plateau time, and at rate of 0.2 per second. The tone bursts were delivered through insert earphones to right and left ears separately, with starting ear randomized and 1–30 Hz EEG filters (Chermak, 1997). In addition, sensitivity/artifact rejection was set at ±100 µV, and the analysis time window opened from −500 to +1,000 ms. The recording wave measures of interest were N1 latency and N1-P2 amplitude, as well as P300 latency and baseline to peak amplitude (Stapells, 2009). Table 3 shows the detailed parameter settings of the LEP recording protocol.

**Table 3:** LEP recording protocol: parameter settings.

Parameter	Setting
Stimulus	1 kHz tone burst (standard)
	2 kHz tone burst (deviant)
	Rise-fall time: 20 ms
	Plateau time: 20 ms
Rate	0.5/s
Polarity	Alternating
Transducers	Insert phones (Ear Tone 3A)
Intensity	60 dB HL (standard)
	90 dB HL(deviant)
Filter	1 to 100 Hz
Analysis time window	−500 to +1,000 ms
Sweeps	100
Sensitivity/artifact reject	±80 µV

In summary, key electrophysiological outcome measures of interest were (1) peak and interpeak latencies for ABR waveforms; (2) N1 latency, and N1 and N1-P2 amplitude; and (3) P300 latency and P300 baseline-peak amplitude.

### Statistical analysis

Descriptive statistics including mean and standard deviation (*SD*) were calculated to present basic characteristics of the current data. An independent *t* test was conducted to compare means of electrophysiological response between genders for both NSCL/P and control groups. Also, the same approach was used to consider possible ear effects in these two groups. In order to investigate differences between children with NSCL/P and craniofacially normal children, two-way ANOVA was used since there were two independent variables—children with and without cleft, and different age groups. Utilizing two-way ANOVA requires that the variance of data are equal for groups of each variable, and the variables in the current study achieved homogeneity of variance except for ABR wave V latency, N1-P2 amplitude and N1 latency. Log transformation was conducted for ABR wave V latency and N1-P2 amplitude to ensure the variances of these two variables were roughly equal. As there was no appropriate transformation approach to improve homogeneity of variance for N1 latency, a non-parametric Mann–Whitney test was conducted to determine whether a significant difference existed between the NSCL/P and control groups. A Kruskal–Wallis test was used to evaluate age effects for both groups. A one-way ANOVA was used to compare N1-P2 amplitude results among the three age categories for NSCL/P and control groups, and the Hochberg test was selected as the post hoc test as group sample sizes were not equal (Field, 2009).

To evaluate cleft type effects among NSCL/P subgroups, a one-way ANOVA was utilized. In order to obtain homogeneity of variance for ABR wave III latency, reciprocal transformation was applied to the data (Field, 2009). Additionally, a non-parametric Kruskal–Wallis test was used for N1 latency results as there was no appropriate transformation method to achieve homogeneity of variance.

In the above analysis, a *p* value <0.05 was considered statistically significant. However, for non-parametric analysis of N1 latency, since comparison was conducted amongst three age groups, three post hoc Mann–Whitney tests were used. In order to control for Type I error, the Bonferroni correction method was employed, and hence the critical value fell to 0.05∕3 = 0.0167 for the N1 latency analysis.

## Results

Descriptive statistics were determined for all electrophysiological results for the overall NSCL/P group and for each cleft subgroup, as well as for the control group (refer to Table 4).

### Comparisons between general NSCL/P and control groups

Comparing electrophysiological results between male and female children with NSCL/P, it was found that on average, ABR wave III (*t*(290) = 4.04, *p* < 0.01, *r* = 0.23), wave V (*t*(290) = 5.62, *p* < 0.01, *r* = 0.31), and wave I–V interpeak latencies (*t*(290) = 5.93, *p* < 0.01, *r* = 0.33) were significantly longer in male than female participants. Also, P300 amplitude of children with cleft showed a significantly larger response in males, *t*(290) = 2.29, *p* < 0.05; however, this was a small sized effect *r* = 0.13. For the control group, significant gender differences were only found in ABR wave III (*t*(118) = 4.41, *p* < 0.01, *r* = 0.38) and wave V latencies (*t*(118) = 5.93, *p* < 0.01, *r* = 0.26). For ear effects, significant differences were not found for any electrophysiological component waveforms, except for N1 latency in the control group, in which left ear latency was significantly longer than right ear latency, *t*(103) = 2.04, *p* < 0.05, *r* = 0.20.

**Table 4:** Electrophysiology results: descriptive statistics for NSCL/P and control groups.

	CL	CP	CLP		NSCL/P	Control group
			UCLP	BCLP		
ABR Wave I Latency (ms)	1.31 ± 0.09	1.36 ± 0.14	1.36 ± 0.11	1.37 ± 0.11	1.35 ± 0.11	1.34 ± 0.09
ABR Wave III Latency (ms)	3.58 ± 0.18	3.67 ± 0.16	3.69 ± 0.17	3.66 ± 0.22	3.65 ± 0.19	3.56 ± 0.15
ABR Wave V Latency (ms)	5.51 ± 0.23	5.60 ± 0.32	5.62 ± 0.22	5.61 ± 0.23	5.58 ± 0.25	5.47 ± 0.19
ABR I–V Interpeak Latency (ms)	4.20 ± 0.22	4.25 ± 0.28	4.26 ± 0.21	4.24 ± 0.21	4.24 ± 0.22	4.13 ± 0.21
N1 Latency (ms)	99.52 ± 24.78	90.31 ± 20.46	102.64 ± 22.54	93.56 ± 29.48	97.95 ± 24.56	77.89 ± 17.30
N1-P2 Amplitude (µv)	9.70 ± 5.34	9.88 ± 5.67	7.82 ± 5.51	9.15 ± 4.58	8.91 ± 5.38	10.85 ± 5.81
P300 Latency (ms)	307.61 ± 35.23	325.18 ± 39.72	306.42 ± 28.67	315.78 ± 43.56	311.83 ± 36.05	325.12 ± 49.52
P300 Amplitude (µv)	5.30 ± 3.69	5.11 ± 3.19	5.31 ± 3.51	4.88 ± 3.54	5.19 ± 3.49	5.51 ± 4.29

**Notes.**

TITLE CL Cleft Lip CP Cleft Palate CLP Cleft Lip and Plate UCLP Unilateral Cleft Lip and Palate BCLP Bilateral Cleft Lip and Plate NSCL/P Non-syndromic Cleft Lip and/or Palate

**Figure 1:**
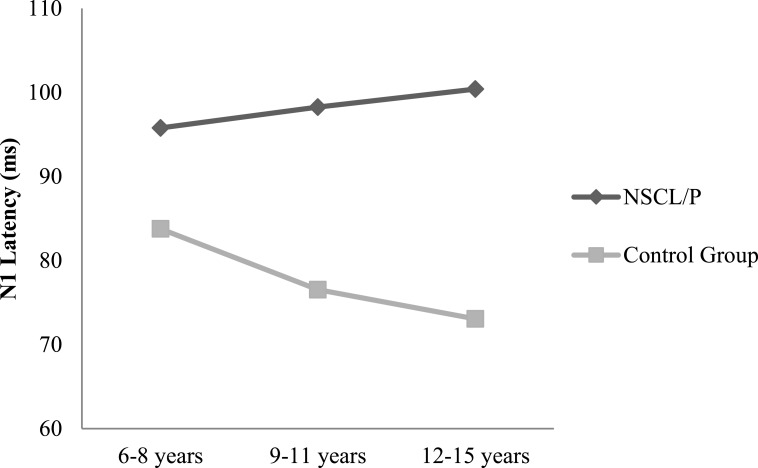
N1 latency changes with age for NSCL/P and control groups.

**Figure 2:**
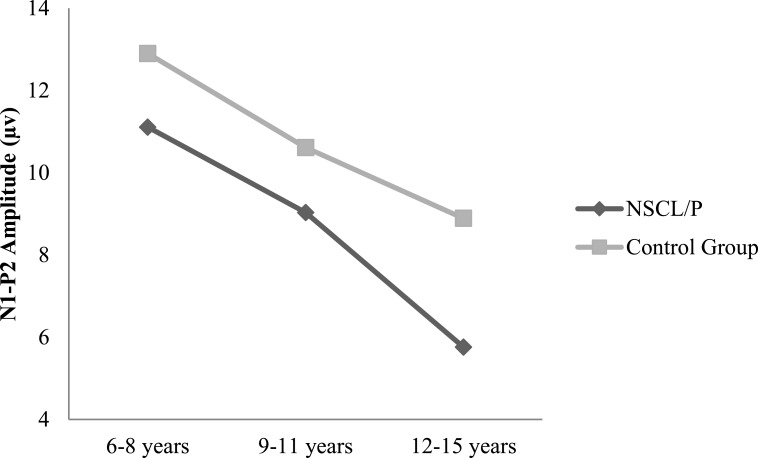
N1-P2 amplitude changes with age for NSCL/P and control groups.

**Figure 3:**
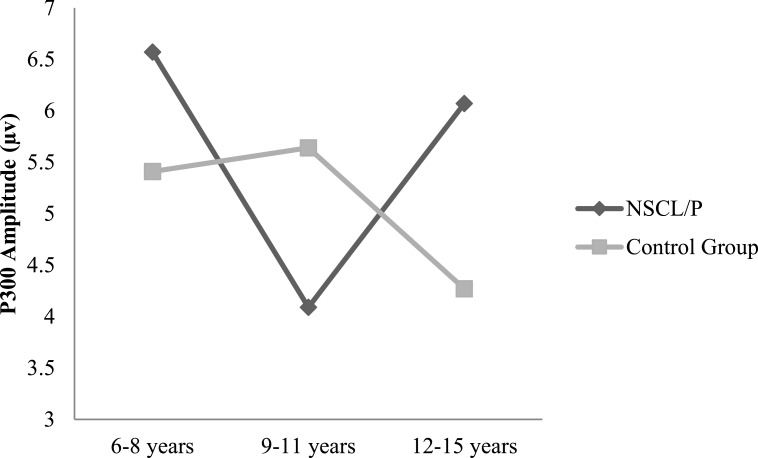
P300 amplitude changes with age for NSCL/P and control groups.

Two-way ANOVA analysis showed that significant differences were found between NSCL/P group and control group for ABR wave III latency (*F*(1, 402) = 21.76, *p* < 0.01), ABR wave V latency (*F*(1, 402) = 20.23, *p* < 0.01), ABR wave I–V interpeak latency (*F*(1, 402) = 16.82, *p* < 0.01), N1-P2 amplitude (*F*(1, 385) = 10.30, *p* < 0.01). Based on Mann–Whitney test results, N1 latency of NSCL/P group was significantly longer than for control group, *U* = 750.5, *z* = −7.59, *p* < 0.01. Kruskal–Wallis test results showed that there was no significant change in N1 latency among different age groups for children with NSCL/P (*H*(2) = 0.69, *p* = 0.71). However, for the control group, N1 latency became significantly shorter with age (*H*(2) = 11.47, *p* < 0.01). Post hoc Mann–Whitney tests showed N1 latency for the craniofacially normal children was significantly different between 6–8 year and 9–11 year groups (*U* = 413.5, *z* = −2.78, *p* < 0.01, *r* = − 0.33), and also between 6–8 year and 12–15 year groups (*U* = 325, *z* = −2.95, *p* < 0.01, *r* = − 0.36). The N1 latency changes in both NSCL/P and control groups with age are shown in Fig. 1. An age effect was also found in both NSCL/P group (*F*(2, 283) = 16.93, *p* < 0.01) and control group (*F*(2, 102) = 3.71, *p* < 0.05) for N1-P2 amplitude, and Hochberg post hoc tests indicated that differences existed between 6–8 year and 12–15 year groups of craniofacially normal children. For NSCL/P, both 6–8 year and 9–11 year groups showed differences compared to 12–15 year group. The age effect on N1-P2 amplitude is shown in Fig. 2. Additionally, the control group did not show any significant difference among age groups for P300 amplitude; however, an age effect was found in NSCL/P group, *F*(2, 285) = 3.88, *p* < 0.05. Hochberg post hoc tests indicated that amplitudes for the 9–11 year group were significantly smaller than for the other two age groups (*p* < 0.05). The age effect on P300 amplitude is shown in Fig. 3.

### Cleft type effects on electrophysiological results in children with NSCL/P

In the current study, in order to evaluate potential differences among subgroups of cleft, children with NSCL/P were divided into four subgroups—cleft lip (CL), cleft palate (CP), unilateral cleft lip and palate (UCLP), as well as bilateral cleft lip and palate (BCLP). Analysis indicated that for ABR wave I, latencies for CP, UCLP and BCLP subgroups were significantly prolonged compared to CL, *F*(4, 408) = 7.24, *p* < 0.01. For ABR wave III latency, significant differences were found between CL vs CP (*F*(4, 408) = 10.23, *p* < 0.05), as well as CL vs UCLP (*F*(4, 408) = 11.59, *p* < 0.01), and longer latencies of ABR wave V were only found in UCLP subgroup compared to CL subgroup (*F*(4, 408) = 8.20, *p* < 0.01). A Kruskal–Wallis test showed UCLP group had significantly longer N1 latency than CP and BCLP subgroup, *H*(4) = 70.52, *p* < 0.01.

## Discussion

### Gender and ear effects on electrophysiological measures

Gender differences found for ABR wave III, V and I–V interpeak latencies in children with NSCL/P, and also for ABR wave III and V latencies of the control group were consistent with typical findings in other studies. It has been consistently reported that, beginning at adolescence, females have slightly shorter wave III and V latencies than males. This difference has been attributed to differences in head size (Sininger & Hyde, 2009). The differences are greatest for wave V, which also results in shorter wave I–V interpeak latencies in females (Jerger & Hall, 1980; Stockard et al., 1979). Similarly, in the current study, the largest ABR differences between genders also existed for wave V. Suggestions have been made that normative data should be separated by gender, since some investigators consider this factor may affect evaluation but others have argued that these differences are not clinically significant (Bauch, Olsen & Pool, 1996; Don & Kwong, 2009). Generally, gender effects for P300 responses are not supported by the research literature, although slightly larger P300 amplitudes have been found for female compared to male normal populations in several studies (Morita et al., 2001; Polich, 1986). On the contrary, the P300 amplitudes of children with NSCL/P were larger in males than females in the current study. However the gender differences were not strong, based on the small effect size noted in the analysis (*r* = 0.13). For ear effects, the only significant difference found was longer N1 latency for the left ear compared to the right ear in the control group in the current study. However, again the effect size was not large (*r* = 0.20). As with gender effects, ear effects have not been raised as an important issue for auditory electrophysiological measures since the differences are too small to be of clinical significance in auditory assessment (Sininger & Hyde, 2009).

### Prolonged ABR wave latencies in children with NSCL/P

ABR has been recommended as an objective assessment tool for hearing screening in infants with CL/P (Arosarena, 2007). An English study utilized ABR to measure the incidence of hearing loss in infants with cleft palate only and found that 82% of the infants tested had hearing loss, and most of them had bilateral mild conductive hearing loss (Viswanathan, Vidler & Richard, 2008). Without consideration of conductive hearing loss, abnormal ABR responses in infants with cleft were rarely found in another study (Yang et al., 2012a). In this latter study infants with middle ear dysfunction and cochlear problems were excluded before ABR testing, and ABR results showed no significant difference between infants with NSCL/P having normal peripheral hearing and their craniofacially normal peers. In the current study, all of the participants who completed electrophysiological tests had passed initial hearing health tests, indicating that although they may have been vulnerable to middle ear dysfunction in their earlier life, such problems had (a) not occurred or (b) resolved with age or appropriate treatment. All assessed children had normal peripheral hearing function at the time they were tested. However, despite any influence of peripheral hearing loss, ABR waves III and V were significantly prolonged in children with NSCL/P compared to their craniofacially normal peers in the current study. In addition, wave I–V interpeak latency was also longer in children with cleft, which may be attributed to the increased wave V latency. Since ABR waves typically achieve adult latency values before 3 years of age these results suggest that, compared to craniofacially normal children, children with cleft may have auditory nerve and/or lower and upper brainstem dysfunction. Prolongation of wave III and V latencies indicated increased neural transmission times between the peripheral auditory nerve and the cochlear nucleus (wave III), and the lateral lemniscus/inferior colliculus (wave V) (Moller, Jannetta & Moller, 1981). Although AEP studies on NSCL/P populations are rarely reported, an analogous study on children with other craniofacial malformations (such as with craniosynostosis) showed similar results to the current research, finding prolongation of ABR wave I–III interpeak latency and III–V interpeak latency (Church et al., 2007).

### Differences in LEP measures

Obligatory components of AEPs such as the P1-N1-P2 complex are noted as exogenous responses since they depend only on external stimulation and change with variation in the physical features of sound, for instance, frequency or intensity (Guzzetta, Conti & Mercuri, 2011). As a result, the P1-N1-P2 complex is free of higher level cognitive confounds such as attention and memory. Compared to the results of the control group in the present study, N1-P1 amplitude was significantly smaller, and N1 latency was prolonged in children with NSCL/P. An increased latency and poorer morphology of N1 was also found in a study which utilized AEPs as an assessment tool for children with suspected auditory processing disorder (Liasis et al., 2003). These investigators suggested the abnormal results indicated slower processing or delayed maturation of the central auditory system within the disorder group. The same reasoning could be applied to the abnormal N1 results in children with NSCL/P in the present study.

In addition, although N1-P2 amplitude gradually decreased in both NSCL/P and control groups, N1 latency only reduced with age in control group children and not in children with cleft disorders. For normal children the P1-N1-P2 complex response continues to undergo changes during maturation until the teenage years (Stapells, 2009). The changes are not limited to decreasing latencies and more robust amplitudes; rather, there are also complex changes in morphology and scalp distribution. Some researchers have demonstrated that small changes in anatomical structure associated with N1 component generator locations result in obvious differences in N1 responses (Tonnquist-Uhlen, Borg & Spens, 1995). As a result, children with craniofacial malformation appear to be at risk of delayed development of the auditory nervous system if the N1 component is considered an index of an impaired system. Also, variation in N1 component maturation among individuals has been attributed to anatomic differences and rate of myelination and synaptogenesis (Sharma et al., 1997). Since auditory processing disorder has been reported relevant to delayed central nervous system myelination, particularly of the corpus callosum, which refers to the brain structure that connects the left and right cerebral hemispheres and facilitates interhemispheric communication (Musiek, Gollegly & Ross, 1985), there are indications emerging from the present study that children with NSCL/P have signs of potential APD, based on their abnormal P1-N1-P2 responses.

P300 has been considered as a cognitive auditory evoked potential, since it often occurs in higher level brain processing associated with stimuli recognition and novelty. Compared to obligatory components of AEPs, P300 has complex generation sites including the primary auditory cortex, frontal cortex and temporal cortex (McPherson, Ballachanda & Kaf, 2007). P300 can reflect the processing abilities for signals using auditory attention and memory after the stimuli arrive at auditory cortical areas. As a result, P300 is elicited by requiring active cooperation from the listener, such as counting the deviant tones of stimuli, and it is often used in the study of memory disorder, information processing, and decision making. To date no known studies have applied P300 in assessment of auditory processing function for children or adults with craniofacial malformation. However, attempts to evaluate individuals with other congenital disorders and cognitive problems using this procedure have been reported. A Brazilian study evaluated latencies and amplitudes of P300 in young adults with Down syndrome, and found prolonged and reduced responses in this population (Cesar et al., 2010). Also, a study on children with attention deficit hyperactivity disorder showed similar abnormal P300 characteristics (McPherson & Davies, 1995). In the current study, there was no significant difference for P300 latency and amplitude between children with NSCL/P and their control group peers, which may suggest that the auditory processing abilities relevant to attention and memory issues available in craniofacially normal children were also present in children with cleft. However, the degree to which processing is available and the quality of the results of processing are not reflected in electrophysiological assessment, and further behavioral evaluation is needed to assess real functional performance in everyday life.

Regarding changes of P300 wave parameters with age in children, a decreasing trend in P300 latency with increasing age, especially between 5 to 12 years has been associated with maturation of cognitive processing function, and latency then begins to increase after about 18 years of age (Stapells, 2009). For the data in the present study, both NSCL/P and control groups did not show significant changes of P300 latency among different age groups. However, for P300 amplitude, age effects were found in children with NSCL/P and these children showed a significantly smaller response in the 9–11 years group. Although the differences for P300 amplitude among age groups in normal children were not statistically significant, there was an apparent anomalous developmental trend in the control group compared to the NSCL/P group, which showed a rising tendency in the younger age NSCL/P group that then reduced in the 12–15 years group (Fig. 3). The control group performance was consistent with the study of Tsai et al. (2012) who investigated age related normative values of AEPs in Taiwanese children. They found P300 amplitude increased in children aged 6–13 years, particularly in children aged 12–13 years, and slightly decreased responses were found after age 13 years leading to normal adult values. Consequently, the findings from the control group in the current study were in agreement with the notion that adolescence is a point of maturation of the P300 AEP component. Based on the findings of an anomalous tendency for age-related P300 amplitude changes in children with NSCL/P, these children may not experience a typical cortical developmental pattern for auditory processing functions.

### Electrophysiological measures for children with NSCL/P: effects of cleft type

In the current study, CL group showed significantly shorter wave latencies than other cleft types for all of ABR wave I, III and V. Conversely, CP group showed significantly longer ABR wave I and III latencies than CL group. Also, prolongation of ABR wave I, III and V latencies were found in UCLP group compared to CL group. In addition, significantly longer N1 latency was also found in UCLP group compared to CP and BCLP groups. Shorter AEP wave latencies imply good maturation of central nervous system development and efficient delivery of auditory input through the auditory nervous pathway. In view of the fact that children with cleft lip only show less maxillofacial malformation than children with cleft palate, this results in a reduced potential incidence of Eustachian tube dysfunction and otitis media. Since receiving reduced auditory stimulation over a long period may influence capacity to process auditory stimuli, children with CL are at lower risk of this problem (Peterson-Falzone, Hardin-Jones & Karnell, 2010), and this may be a reason why CL group had more robust AEPs than other cleft types. Alternatively, a greater degree of craniofacial malformation (such as found in CP and CLP groups) may more directly relate to greater risk of cortical abnormalities. Prolonged ABR wave latencies for CP group indicated longer transmission times between peripheral auditory nerve and the cochlear nucleus in this group. Furthermore, the abnormal results for both ABR waves and N1 latencies obtained from UCLP group suggested slower speed of nervous activities from neural structures peripheral to the auditory midbrain (Eggermont, 2007), as well as possibly delayed development of myelination and synaptogenesis (Sharma et al., 1997). In a majority of studies, children with CL and children with CL/P are often pooled together, since an assumption is made of a common genetic origin for these two cleft types (McCarthy, Curring & Hogan, 1990). However, in addition to the present study, one previous report—which utilized MMN to evaluate auditory short-term memory for children with oral clefts—also found weaker responses in a UCLP group compared to a CL group, and the more posteriorly delimited the cleft was, the poorer were the MMN responses (Ceponiene et al., 1999). Hence, separation of CL and CLP groups is recommended for electrophysiological studies of APD in children with cleft disorders.

## Conclusion

Based on the above results, children with NSCL/P may have slower than normal neural transmission times between the peripheral auditory nerve and the brainstem. Furthermore, delayed development of myelination and synaptogenesis may also adversely influence auditory processing function in this population. In addition, electrophysiological performance was noted to be related to cleft type and it is important to consider different cleft types separately when electrophysiological research is conducted for children with NSCL/P. This study may enhance awareness of auditory processing issues in children with cleft disorders, and draw attention to the potential functional problems associated with these APD issues—problems that have also been noted in behavioral and questionnaire studies with NSCL/P children. In future electrophysiological studies, use of a multiple-channel recording system for evoked response potentials is suggested to obtain more detailed insights into the auditory cortical development of school age children with NSCL/P.

##  Supplemental Information

10.7717/peerj.2383/supp-1 Data S1 Raw data NSCLP Children

10.7717/peerj.2383/supp-2 Data S2 Raw data for control children

## Additional Information and Declarations

### Data Availability

The following information was supplied regarding data availability: The raw data has been supplied as Supplemental Information.
